# Optogenetic elevation of postsynaptic cGMP in the hippocampal dentate gyrus enhances LTP and modifies mouse behaviors

**DOI:** 10.3389/fnmol.2024.1479360

**Published:** 2024-11-26

**Authors:** Jelena Borovac, Jayant Rai, Megan Valencia, Hang Li, John Georgiou, Graham L. Collingridge, Keizo Takao, Kenichi Okamoto

**Affiliations:** ^1^Lunenfeld-Tanenbaum Research Institute, Mount Sinai Hospital, Toronto, ON, Canada; ^2^Department of Molecular Genetics, Faculty of Medicine, University of Toronto, Toronto, ON, Canada; ^3^Department of Behavioral Physiology, Graduate School of Medicine and Pharmaceutical Sciences, University of Toyama, Toyama, Japan; ^4^Department of Physiology, Faculty of Medicine, University of Toronto, Toronto, ON, Canada; ^5^TANZ Centre for Research in Neurodegenerative Diseases (CRND), University of Toronto, Toronto, ON, Canada; ^6^Department of Behavioral Physiology, Faculty of Medicine, University of Toyama, Toyama, Japan; ^7^Research Centre for Idling Brain Science, University of Toyama, Toyama, Japan

**Keywords:** cGMP, optogenetics, electrophysiology, long-term potentiation, synaptic plasticity, memory, mouse behaviors

## Abstract

A major intracellular messenger implicated in synaptic plasticity and cognitive functions both in health and disease is cyclic GMP (cGMP). Utilizing a photoactivatable guanylyl cyclase (BlgC) actuator to increase cGMP in dentate granule neurons of the hippocampus by light, we studied the effects of spatiotemporal cGMP elevations in synaptic and cognitive functions. At medial perforant path to dentate gyrus (MPP-DG) synapses, we found enhanced long-term potentiation (LTP) of synaptic responses when postsynaptic cGMP was elevated during the induction period. Basal synaptic transmission and the paired-pulse ratio were unaffected, suggesting the cGMP effect on LTP was postsynaptic in origin. In behaving mice implanted with a fiber optic and wireless LED device, their performance following DG photoactivation (5–10 min) was studied in a variety of behavioral tasks. There were enhancements in reference memory and social behavior within tens of minutes following DG BlgC photoactivation, and with time (hours), an anxiogenic effect developed. Thus, postsynaptic cGMP elevations, specifically in the DG and specifically during conditions that evoke synaptic plasticity or during experience, are able to rapidly modify synaptic strength and behavioral responses, respectively. The optogenetics technology and new roles for cGMP in the DG may have applications in brain disorders that are impacted by dysregulated cGMP signaling, such as Alzheimer’s disease.

## Introduction

The intracellular messengers, cyclic AMP (cAMP) and cyclic GMP (cGMP), are involved in the mechanisms of synaptic plasticity and memory, and their modulation is being considered as treatments for various disorders including Alzheimer’s disease (AD) (Zhuo et al., 1994; Zhuo and Hawkins, 1995; Borovac et al., 2018; Ricciarelli and Fedele, 2018; Jehle and Garaschuk, 2022). The medial perforant path to dentate gyrus (MPP-DG) synapses exhibit postsynaptic NMDA receptor-dependent LTP and are critical for learning and memory (Bliss and Collingridge, 1993; Moser et al., 1998). Postsynaptic cAMP plays a key role in the protein synthesis-dependent long-term potentiation (L-LTP) at the MPP-DG synapses (Nguyen and Kandel, 1996). Utilizing an optogenetic approach to manipulate cAMP by light in the hippocampal DG granule neurons of mouse brain slices, we have shown that cAMP has the capacity to facilitate LTP at MPP-DG synapses and to increase the spread of neuronal depolarization (Luyben et al., 2020).

However, the dynamic mechanisms by how cGMP contributes to the induction of NMDA receptor-dependent LTP at the MPP-DG synapses and hippocampal DG-dependent memory are still not well-known. NMDA receptor stimulation leads to activation of a nitric oxide (NO)-dependent cGMP pathway through calcium–calmodulin (Ca^2+^/CaM) stimulation of neuronal nitric oxide synthase (nNOS) (Mayer et al., 1990; Mayer et al., 1992; Chalimoniuk et al., 1996). The resultant cGMP activates its major effectors including cGMP-dependent protein kinase (PKG), phosphodiesterases (PDEs), and cyclic nucleotide gated-ion channel (CNG) (Nolan et al., 2004; Feil and Kleppisch, 2008; Francis et al., 2010; Sanderson and Sher, 2013; Borovac et al., 2018). Presynaptic cGMP, synthesized by retrograde NO activation of the guanylyl cyclase enzyme (GC), is known to facilitate presynaptic vesicle release (Feil and Kleppisch, 2008). However, the role of postsynaptic cGMP in LTP at MPP-DG synapses is not well understood.

cGMP function in memory has been addressed through pharmacological inhibition of phosphodiesterase (PDE) enzymes, which degrade cGMP, cAMP, or both (Bender and Beavo, 2006). PDE2 hydrolyzes cAMP and cGMP, while PDE5 and PDE9 are cGMP-specific (Blokland et al., 2006). Remarkably, PDE2 (Boess et al., 2004), PDE5 (Prickaerts et al., 2004), and PDE9 (van der Staay et al., 2008) inhibition have all been reported to significantly improve learning and memory in rodents. These findings suggest that upregulation of cGMP signaling is associated with improved memory. Furthermore, an inhibitor of PDE5 was reported to reverse cognitive dysfunction in AD rodent models, indicating potential cGMP impairments in neurodegenerative diseases (Puzzo et al., 2009; Cuadrado-Tejedor et al., 2011; Garcia-Barroso et al., 2013). Indeed, AD and depressed patients have been found to have reduced cGMP levels, with the degree of reduction correlating with the extent of cognitive impairment (Ugarte et al., 2015; Hesse et al., 2017).

To directly determine the function of cGMP signaling, we virally expressed a bacterial light-activated guanylyl cyclase (BlgC) (Ryu et al., 2010) in murine DG granule neurons. We examined synaptic responses in the acutely prepared hippocampal slices by electrophysiology. Photostimulation of the cGMP actuator (BlgC) during MPP-DG pathway tetanic stimulation led to an enhancement of LTP. The findings demonstrate that transiently enhancing cGMP signaling during MPP activity has the capacity to enhance synaptic efficacy.

Using a wireless LED device with fiber optics implanted into the mouse brain, we next analyzed the behavioral phenotypes of mice following photostimulation of DG BlgC (5–10 min) during a comprehensive battery of behavioral tests. Within minutes, the cGMP group showed enhanced reference memory and sociability, and with time (2 h), an anxiogenic effect developed. The findings demonstrate that transiently enhanced cGMP in DG granule neurons can enhance synaptic plasticity and modify mouse behaviors.

## Materials and methods

### Animals

Acute hippocampal slices, stereotaxic surgery for viral injection, and LED implant protocols and animal procedures for mouse behavioral assays were approved by the animal care committees at The Centre for Phenogenomics (TCP; Toronto, ON, Canada) and University of Toyama (Japan). Organotypic slice cultures of hippocampus were prepared from postnatal day 6–7 Sprague Dawley rat pups as previously described (Stoppini et al., 1991; Okamoto et al., 2004) and in accordance with the guidelines of the University Health Network animal care committee approved protocol and oversight (TCP, Canada).

### Expression vectors

BlgC (Ryu et al., 2010): human codon-optimized with S27A mutation to reduce dark activity (Stierl et al., 2014) was fused with RFP (tdTomato) (Shaner et al., 2004) at the N-terminus and subcloned into pCAG plasmid vectors (Niwa et al., 1991). The cGMP FLIM probe (cGiYR: cGMP indicators with YFP/RFP) was constructed from the Gln^79^—Tyr^345^ in cGMP-dependent protein kinase I (cGKI) (Russwurm et al., 2007) fused with YFP (Citrine) (Heikal et al., 2000) as a FRET/FLIM donor and RFP (tdTomato) as a FRET/FLIM accepter into pCAG plasmid vector. The cAMP FLIM probe (REY: RFP-Epac-YFP) (Luyben et al., 2024), and a control YFP (Citrine) were also subcloned into pCAG plasmid vectors.

### Viruses

AAV1-CaMKIIα promoter-mGFP-BlgC (titer: 2.07 × 10^13^ VG/mL for behavioral assays, 2.11 × 10^13^ VG/mL for electrophysiology) was packaged and purified by SignaGen laboratories (SL100863 and SL100864, Rockville, MD, United States).

### Detection of BlgC photoactivation by two-photon FLIM

For *in vitro* cGMP detection by two-photon FLIM, HEK293 cell lysates expressing cGMP FLIM probe (cGiYR) were placed in a microscope chamber and we measured the dose-dependent lifetime change by serially adding 8-Br-cGMP and comparing with a control cell lysate which express YFP alone using a two-photon FLIM microscope equipped with a time-correlated single photon counting (TCSPC) system (PicoHarp 300, FLIM upgrade kit for Olympus FV1000MPE, SymPhoTime software; PicoQuant, Berlin, Germany) using a single plane of YFP fluorescence (520–542 nm) images (donor of cGiYR: 900 nm excitation, 30-s repetitive FLIM imaging time) (Luyben et al., 2024). Fluorescence lifetime data were analyzed by SymPhoTime software (PicoQuant, Berlin, Germany) (Hille et al., 2009). For average time-domain fluorescence lifetime measurements, the lifetime decay curves were fit by a double exponential decay model using the tail-fitting analysis. To detect BlgC photoactivation in living neurons, organotypic hippocampal slice cultures were prepared (Okamoto et al., 2004) and biolistically transfected cGMP FLIM probe cGiYR with or without BlgC (or cAMP FLIM probe REY with BlgC as a control). The expressed BlgC was photoactivated by single photon light (414 ± 21 nm, mercury arc lamp, 60 s) in the neurons. The lifetime change of the FLIM probes were measured by two-photon FLIM microscopy as described above for the *in vitro* experiments. The fluorescence lifetime pseudo-color image of neurons was constructed using a pixel by pixel fitting function.

### Stereotaxic AAV microinjection

Next, 14- to 16-week-old C57BL/6J male mice were anesthetized with isoflurane (2% vol/vol) and then transferred to a stereotaxic apparatus. Mice were secured to the apparatus by fixing ear bars to the head and inserting the incisor adaptor. A nose cone was used to administer isoflurane during surgery to maintain anesthesia as confirmed by mild pinching of the tail. Mice were injected with 0.1 mL of meloxicam (0.5 mg/mL) and 0.1 mL of saline subcutaneously prior to surgery. Hair atop the scalp was removed with electric clippers, and antiseptic solution was applied topically to clean the skin. In addition, Tear-Gel (Alcon, TX, United States) was applied to lubricate the eyes and reapplied when necessary. A scalpel was used to make a midline incision and expose the bregma and lambda landmarks of the skull. After leveling the head, a drill was used to make two small holes 2 mm posterior from bregma and 1.3- or 1.6-mm lateral to the midline. Injection volumes were 2 μL per hemisphere and delivered at a rate of 0.1–0.2 μL per min using a gastight syringe with a 26-gauge needle (Hamilton, Reno, NV, United States). Following injection, the syringe was kept in place for an additional 5 min to allow diffusion of the virus particles. The syringe was then slowly withdrawn from the head, and the injection site was wiped clean. The skin was sutured, and antibiotic ointment was applied over the wound. The mouse was removed from the stereotaxic apparatus and placed in a recovery cage on a heat pad. Meloxicam was administered subcutaneously every 24 h for 2 to 3 days following surgery. Mice were given at least 3 weeks of postoperative recovery. For validation of the expression, mouse brain coronal sections were prepared and imaged with DAPI staining as previously described (Yokose et al., 2017).

### Electrophysiology

Acute hippocampal slices were prepared as previously described (Luyben et al., 2020) from adult BlgC-expressing mice and their wild-type littermates. After 1.5- to 2-h recovery, slices were transferred to a chamber perfused with an ACSF solution containing 124 mM NaCl, 3 mM KCl, 2.5 mM CaCl_2_, 1.3 mM MgSO_4_, 1.25 mM NaH_2_PO_4_, 26 mM NaHCO_3_, and 10 mM glucose (pH 7.4, 30°C, 1.5 mL/min) equilibrated with 5% CO_2_/95% O_2_. Recordings of field excitatory postsynaptic potentials (fEPSPs) were recorded as previously described (Luyben et al., 2020) in the DG while blocking inhibitory synaptic function using 10 μM bicuculline (Saab et al., 2009). The stimulation electrode was positioned in the dorsal blade of the dentate molecular layer for MPP stimulation. Paired field responses were evoked by stimulating with an intensity (0.05 ms pulses, 40 ms apart) that yielded fEPSPs that were 40% of the maximum spike-free fEPSP size. Responses were evoked and acquired every 20 s throughout the experiment using an Axopatch 1D amplifier (Axon Instruments) digitized at 20 kHz and measured by slope (10–50% of fEPSP rising phase). The expressed BlgC in DG granule cells was photoactivated using a blue LED light: 1.5 mW, 4 min for baseline synaptic responses, 5 min for LTP (4 min before tetanus and 1 min during tetanus) under an objective lens (4X, NA0.1). Tetanus was induced with a bipolar tetanic stimulation (100 Hz, 0.15 ms pulses delivered in 4 trains of 0.5 s duration, 20 s apart). In the time course experiments, field responses were plotted by normalizing to the baseline fEPSP slope (average of the 10-min period prior to tetanic stimulation).

### cGMP photoactivation assay by ELISA

The hippocampal slices from BlgC and WT littermate mice were homogenized in buffer (40 mM HEPES/Na, pH 8.0, 0.1 mM EGTA, 5 mM magnesium acetate, 1 mM DTT, and 0.01% Tween-20) by sonication and centrifuged at 16,000 g for 15 min to clear large tissue debris as previously described (Luyben et al., 2020). The expressed hippocampal lysates were photoactivated for 5 min with a 455 nm LED (4.5 mW/mm^2^; ThorLabs, NJ, United States) on a plastic paraffin film (Parafilm M^®^, Bemis, United States) covered glass slide at room temperature in the dark with 200 μM GTP. After photoactivation, the reactions were stopped by application of 0.1 M HCl following the ELISA kit protocol. The synthesized cGMP concentrations were measured by a cGMP ELISA kit (Enzo Life Sciences, NY, United States).

### LED implant

For behavioral testing, C57BL/6J male mice with BlgC AAV injection were surgically equipped with a bilateral LED (or dummy) implant targeting the same site where virus was injected. A bilateral 470 nm wavelength LED device (0.75 mm diameter, 20 mW, Eicom USA, CA, United States) was inserted slowly to minimize damage to brain tissue. The LED device was fixed to the skull using two kinds of dental cement (Bistite II DC, Tokuyama Dental America Inc., Japan, and UNIFAST Trad Liquid, GC America Inc., United States) and cured. The mouse was then removed from the stereotaxic device and placed in a recovery cage on a heat pad (the implant work was done in Japan). Analgesia (METACAM) was continued by subcutaneous injection every 24 h for 3 days post-surgery. At least 14 days were interspaced between surgery and behavioral testing.

### Mouse behavior

Mice were surgically equipped with either a bilateral fiber optic LED system (Eicom USA, CA, United States) or, as a control, a “dummy” optic fiber, which produces no light but exhibits the same physical properties. The mice were housed in a home cage monitoring system with automated video tracking and habituated to wearing a detachable battery (necessary to power the LED). The battery (or dummy battery) was attached and turned on to produce cGMP during each test (light-activation details available in each figure). All behavioral tests were performed as previously described (Ageta-Ishihara et al., 2013; Shoji et al., 2016). The control and BlgC mouse cohorts were counterbalanced such that each test was performed on equal number of control and experimental mice simultaneously (in the case of multiple chambers). In single chamber tests, a control mouse test was followed by BlgC mouse test until all mice were evaluated. After the behavior testing was complete, the mice were sacrificed and checked for the expression of BlgC.

#### General health and neurological screen

The general health and neurological screen (GHNS) were conducted as previously described (Shoji et al., 2016). Body weight and rectal temperature were measured. Neuromuscular strength was assessed using the grip strength and wire hang tests. A grip strength meter (O’Hara & Co., Tokyo, Japan) was used to assess forelimb grip strength. Mice were lifted and held by their tail so that their forepaws could grasp a wire grid. The mice were then gently pulled backward by the tail until they released the grid. The peak force applied by the forelimbs of the mouse was recorded in Newton (N). Each mouse was tested three times, and the largest value was used for statistical analysis. In the wire hang test, the mouse was placed on a wire mesh that was then inverted, and the latency to fall from the wire was recorded with a 60-s cutoff time.

#### Rotarod test

Motor coordination and balance were tested with a rotarod (RR) test. The rotarod (RR) test, (UGO Basile Accelerating Rotarod, Varese, Italy) was performed by placing mice on accelerating rotating drums (3 cm diameter) and measuring the time each animal was able to maintain its balance on the rod. The speed of the rotarod accelerated from 4 to 40 rpm over a 5-min period. All mice were fitted with a battery for 5 min immediately prior to the screening with the light turned on to generate cGMP in BlgC mice.

#### Hot plate test

The hot plate (HP) test was performed to evaluate sensitivity to painful stimuli. Mice were placed on a 55.0 ± 0.3°C hot plate (Columbus Instruments, Columbus, OH, United States), and latency to the first paw response was recorded with a 15-s cutoff time. The paw response was defined as either a foot shake or a paw lick. All mice were fitted with a battery for 5 min immediately prior to the screening with the light turned on to generate cGMP in BlgC mice.

#### Light/dark test

The light/dark (LD) transition test apparatus consisted of a cage (21 × 42 × 25 cm) divided into two sections of equal size by a partition with a door (O’Hara & Co., Tokyo, Japan). One chamber was brightly illuminated (390 lux), whereas the other chamber was dark (2 lux). Mice were placed into the dark chamber and allowed to move freely between the chambers with the door open for 10 min. The total number of transitions between chambers, time spent in each chamber (s), latency to first enter the light chamber (s), and distance traveled in each chamber (cm) were recorded automatically by ImageLD software. All mice were fitted with a battery for 5 min immediately prior to the test with the light turned on to generate cGMP in BlgC mice.

#### Open field test

The open field (OF) test apparatus was a transparent square cage (42 × 42 × 30 cm; Accuscan Instruments, Columbus, OH, United States). The center of the floor was illuminated at 100 lux. Each mouse was placed in the open field apparatus and recorded for 120 min. The total distance traveled (cm) and time spent in the center area (20 × 20 cm) were measured. Due to the longer testing time in the OF (120 min) compared with other behavioral tests (10–15 min), all mice received 10 min of light activation to generate cGMP in BlgC mice.

#### Elevated plus maze

The elevated plus (EP) maze consisted of two open arms (25 × 5 cm, with 3-mm-high ledges) and two closed arms (25 × 5 cm, with 15-cm-high transparent walls) of the same size (O’Hara & Co., Tokyo, Japan). The arms and central square were made of white plastic plates and were elevated to a height of 55 cm above the floor. The arms of the same type were arranged on opposite sides to each other. The center of the maze was illuminated at 100 lux. Each mouse was placed in the central square of the maze (5 × 5 cm), facing one of the closed arms, and was recorded for 10 min. The distance traveled (cm), number of total entries into arms, percentage of entries into open arms, and percentage of time spent in open arms were calculated automatically using ImageEP software. All mice were fitted with a battery during the test with the light turned on for the first 5 min to generate cGMP in BlgC mice. Two datasets of BlgC mice were missing due to technical issues.

#### Social interaction test

Two mice from the cohort (paired as BlgC and control, whenever possible) were placed into a box together (40 × 40 × 30 cm; O’Hara & Co., Tokyo, Japan) and were allowed to explore freely for 10 min. Mouse behavior was analyzed automatically using ImageSI software. The total duration of contacts (sec), number of contacts, total duration of active contacts (sec), mean duration per contact, and total distance traveled (cm) were measured. The active contact was defined as follows: Images were captured at three frames per sec, and the distance traveled between two successive frames was calculated for each mouse. If the two mice contacted each other and the distance traveled by either mouse was 5 cm and more, the behavior was considered an “active contact.” All mice were fitted with a battery during the test with the light turned on for the first 5 min to generate cGMP in BlgC mice.

#### Crawley’s social interaction test

The Crawley’s social interaction (CSI) test apparatus had three chambers (20 × 40 × 22 cm) separated by two transparent partitions each with an opening (5 × 3 cm) and a lid with an infrared CCD camera. A male mouse (8–12 weeks old C57BL/6J, termed Stranger 1) that had no prior contact with the subject mice was enclosed in a cylinder cage (9 cm ϕ, set in the left chamber) that allowed nose contacts. The subject mouse was released in the middle chamber and allowed to explore for 10 min, while the time spent in each chamber and within 5 cm from each cage was measured automatically using ImageCSI software. Subsequently, another unfamiliar mouse (Stranger 2) was placed in another cylinder cage (in the right chamber) and monitored likewise for another 10 min. All mice were fitted with a battery for 5 min immediately prior to the test with the light turned on to generate cGMP in BlgC mice.

#### Object location test

First, the mice were placed in a solid plastic chamber (30 × 30 × 30 cm) and allowed to habituate for 10 min a day for 3 days before test. For the familiarization phase, the mice were placed in a solid plastic chamber (30 × 30 × 30 cm) containing two identical objects, equidistant from the center and walls, placed in opposite corners. After 15 min, mice were placed back into their home cage. On the next day, the mice returned to the same chamber where one of the objects was moved to a novel location and allowed to explore for 15 min. In all the experiments, the same objects (a cube with black dots) were used, and data for the time around each object (sec) and number of entries (based on the selected region of interest) were collected. Data acquisition and analysis were performed automatically using ImageOF. All mice were fitted with a battery for 5 min during familiarization phase with the light turned on to generate cGMP in BlgC mice. Approaches to the objects for the first 5 min of the test session were analyzed.

#### Barnes maze

The Barnes circular maze task was conducted on a white circular surface (1.0 m in diameter, with 12 holes equally spaced around the perimeter; O’Hara & Co., Tokyo, Japan). The circular open field was elevated 75 cm from the floor. A black Plexiglas escape box (17 × 13 × 7 cm), which had paper cage bedding on its bottom, was located under one of the holes. The hole above the escape box represented the target, analogous to the hidden platform in the Morris water maze task. The location of the target was consistent for a given mouse but randomized across mice. The maze was rotated daily, with the spatial location of the target unchanged with respect to the distal visual room cues, to prevent a bias based on olfactory or proximal cues within the maze. Mice were trained for 15 trials, with three training trials per day for 5 days. A single training trial was conducted immediately after each probe test. All mice in BlgC and control groups received the same number of training sessions on the same days. The number of errors, latency to reach the target (s), and distance traveled to reach the target (cm) were automatically calculated by ImageBM software.

Twenty-four hours after the last training, a probe test trial (PT1) was conducted without the escape box for 3 min, to confirm that this spatial task was acquired based on navigation by distal environment cues (memory retrieval). After PT1, one training trial was performed to make subjects recognize the escape box again. The second probe test trial (PT2) was conducted 2 weeks after the last training to evaluate memory retention. The time spent around each hole, number of errors, moving time (s), distance traveled (cm), and moving speed (cm/s) were recorded using ImageBM software. All mice were fitted with a battery for 5 min immediately prior to each training session (original training and reversal training) with the light turned on to generate cGMP in BlgC mice.

#### T-maze (forced alternation)

The forced alternation task was conducted using an automatic T-maze (O’Hara & Co., Tokyo, Japan) constructed of white plastics runways with walls 25-cm high as previously described (Takao et al., 2008). The maze was partitioned off into six areas by sliding doors that can be opened downward. The stem of T was composed of area S2 (13 × 24 cm), and the arms of T were composed of areas A1 and A2 (11.5 × 20.5 cm). Areas P1 and P2 were the connecting passageway from the arm (areas A1 or A2) to the start compartment (area S1). The end of each arm was equipped with a pellet dispenser that could provide food reward. The pellet sensors were able to record automatically pellet intake by the mice. One week before the pre-training, mice were deprived of food until their body weight was reduced to 80–85% of the initial level. Mice were kept on a maintenance diet throughout the course of all the T-maze experiments. Before the first trial, mice were subjected to three 10-min adaptation sessions, during which they were allowed to freely explore the T-maze with all doors open and both arms baited with food. One day after the adaptation session, mice were subjected to a forced alternation protocol for 5 sessions (one session consisting of 10 trials per day; cutoff time, 50 min). On the first (sample) trial of each pair, the mouse was forced to choose one of the arms of the T (areas A1 or A2) and received the reward at the end of the arm. Choosing the incorrect arm resulted in no reward and confinement to the arm for 10 s. After the mouse consumed the pellet or the mouse stayed more than 10 s without consuming the pellet, door that separated the arm (area A1 or A2) and connecting passageway (area P1 or P2) would be opened and the mouse could return to the starting compartment (area S1), via connecting passageway, by itself. The mouse was then given a 3-s delay and a free choice between both T arms and rewarded for choosing the other arm that was not chosen on the first trial of the pair. The location of the sample arm (left or right) was varied pseudo-randomly across trials using Gellermann schedule so that mice received equal numbers of left and right presentations. On the 16–21st day, delay (10, 30, or 60 s) was applied after the sample trial. Data acquisition, control of sliding doors, and data analysis were performed by Image TM software. All mice were fitted with a battery for 5 min immediately prior to each training session (consisting of 10 L/R trials) with the light turned on to generate cGMP in BlgC mice. Four datasets of BlgC mice were excluded due to technical issue and death during the experiment.

### Statistical analysis

Statistical methods are indicated in the figure legends. All data are presented as mean ± SEM. ***p < 0.001; **, *p* < 0.01; *, *p* < 0.05; NS, *p* > 0.05.

## Results

### Expression and photoactivation of BlgC

**Figure 1:**
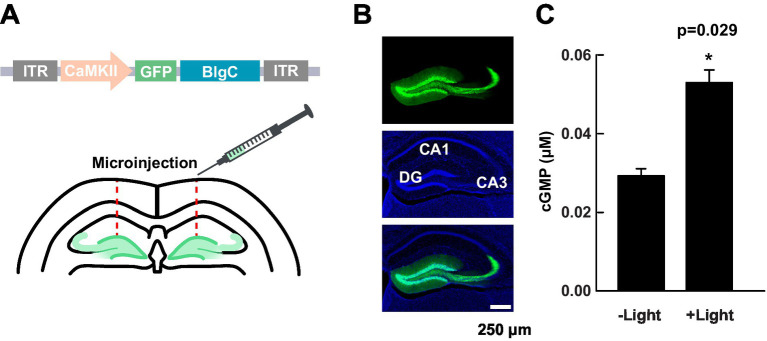
Expression and validation of BlgC in DG neurons. **(A)** Schematic of the transfer plasmid components within the inverted terminal repeats (ITR); mice received bilateral DG injections of AAV1-CaMKIIα promoter-GFP-BlgC. **(B)** A representative image of the distribution of GFP-BlgC in hippocampal slices. GFP fluorescence (green, upper), DAPI staining (blue, middle), and merged (lower) images collected by a confocal microscope. **(C)** Measurement of cGMP in hippocampal slice lysate by ELISA following either photoactivation (+Light) of BlgC or no (−) light control. The illumination was carried out by 455 nm LED (4.5 mW/mm^2^ for 5 min, *n* = 3, unpaired *t*-test). Data are presented as mean ± SEM.

The study was designed to determine the role of cGMP in synaptic plasticity and cognitive functions, with a specific focus on MPP fibers synapsing onto dentate gyrus (DG) granule neurons (MPP-DG synapses). Bilateral DG injections of virus expressing a green fluorescence protein (GFP)-tagged photoactivatable BlgC (Ryu et al., 2010) under the CaMKIIα promoter were carried out, and the expression was detected in granule cells including their mossy fiber projections to CA3 (Figures 1A,B). We know that the BlgC actuator system generates cGMP since we tracked its dynamics within neurons from organotypic hippocampal slices in cultures. Specifically, we introduced a cGMP FLIM probe, cGiYR (cGMP indicators with YFP/RFP cGMP FLIM) and, upon blue light stimulation (414 ± 21 nm), determined that the fluorescence lifetime of cGiYR increased and was maintained for at least 10 min, specifically in the neurons biolistically co-transfected both BlgC and cGiYR probe, but not cGiYR probe alone or REY (cAMP probe) + BlgC (Supplementary Figure S1). We then returned to the mouse brain to evaluate whether the BlgC-expressing DG neurons can produce a cGMP response. Hippocampal slice lysate was photostimulated (455 nm LED, 4.5 mW/mm^2^, for 5 min), and subsequently, cGMP levels were determined by ELISA. Compared with the no-light control, the photoactivated group showed elevated cGMP levels, thus demonstrating enzymatic functionality and effective application of the BlgC actuator system within the DG (Figure 1C).

### Expression of BlgC does not affect baseline MPP-DG synaptic transmission

**Figure 2:**
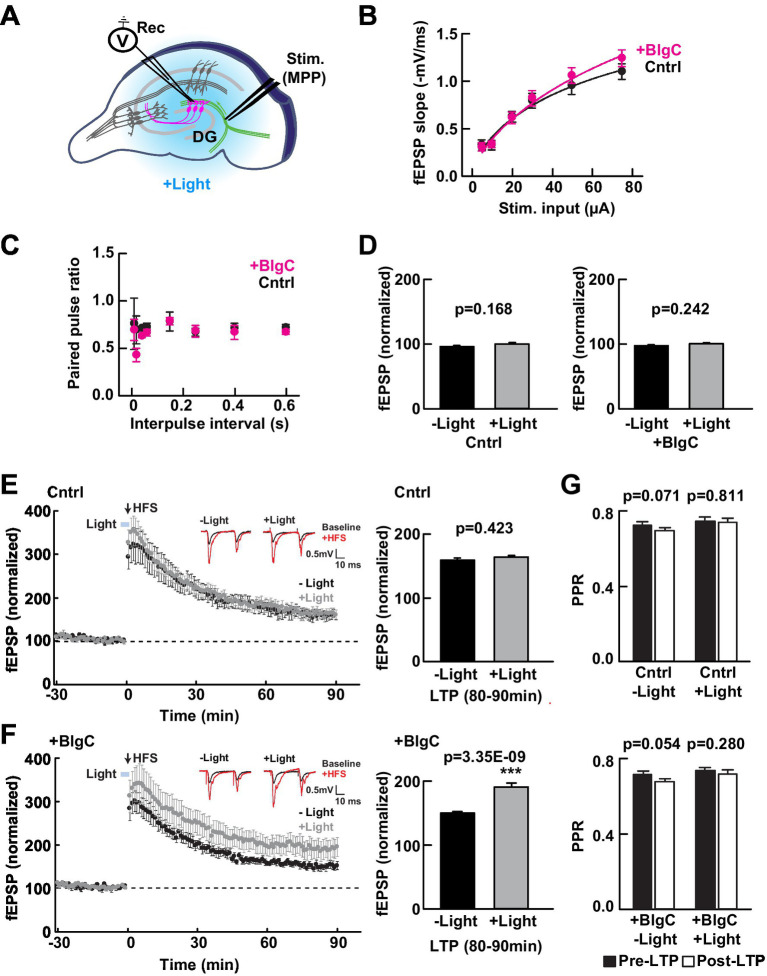
Photoactivation of BlgC enhances long-term potentiation (LTP) at medial perforant path to dentate gyrus (MPP-DG) synapses. **(A)** Schematic of the DG recording (Rec) electrode to detect fEPSPs upon stimulation (stim) of the MPP in acutely prepared hippocampal brain slices. Light was delivered for photoactivation (blue light, 480 ± 15 nm) of BlgC expressed within DG granule neurons (magenta). **(B)** Input/output relationship for the wild-type (black, *n* = 11 slices/10 mice) and BlgC group (magenta, *n* = 14 slices/12 mice). **(C)** Comparison of paired-pulse ratio at MPP-DG synapses between wild-type and mice expressing BlgC within the DG. Paired-pulse ratio was calculated from the slope of the second fEPSP response slope divided by that of the first, delivered at different various interpulse intervals as shown (control *n* = 6 slices/6 mice, BlgC *n* = 6 slices/6 mice). **(D)** Quantification of the baseline synaptic response with and without photoactivation. Slices were photostimulated (480 ± 15 nm, 1.5 mW, 4 min) during baseline recording and compared before (-Light) and 4 min after light stimulation (+Light) under a fluorescence microscope (left: control (Cntrl) *n* = 6 slices/6 mice, right: BlgC (+BlgC) *n* = 6 slices/6 mice, unpaired t-test). **(E)** Time course of LTP induced by high frequency stimulation (HFS) in control mice with (+Light, Cntrl; grey; *n* = 6 slices/6 mice) and without (-Light, Cntrl; black; *n* = 6 slices/6 mice) illumination by blue light (480 ± 15 nm, 5 min, blue bar). LTP was compared as the average of synaptic response (fEPSP) slope at 80-90 min after tetanic stimulation (bar graph, right) and showed no significant effect of light on LTP in control mice. *n* = 6 slices/6 mice. Paired *t*-test. Inset: sample paired-pulse traces (scale bar of 0.5 mV and 10 msec) show superimposition of the fEPSP response before (Baseline; black) and one min after tetanic stimulation (+HFS; magenta). **(F)** LTP in hippocampal slices expressing BlgC in dentate granule neurons, with (+Light, +BlgC; grey; *n* = 6 slices/6 mice) and without (-Light, +BlgC; black; *n* = 6 slices/6 mice) illumination by blue light (480 ± 15 nm, 5 min, blue bar). There was a significant enhancement of LTP in the light-stimulated condition (+BlgC, LTP (80-90 min); paired *t*-test). All data are mean ± SEM. Inset: sample paired-pulse traces (scale bar of 0.5 mV and 10 ms) show superimposition of the fEPSP response before (baseline; black) and one min after tetanic stimulation (+HFS; magenta). **(G)** Comparison of paired-pulse ratio before (-10 to 0 min, black, pre-LTP) and after tetanic stimulation (80 to 90 min, white, post-LTP) ± blue light in both control (Cntrl: upper) and DG BlgC-expressing hippocampal slices (+BlgC: lower). paired t-test). All data are mean ± SEM.

To test whether the expression of BlgC (without photoactivation) has any impact on the basal synaptic transmission at intact MPP-DG synapses, we measured the field excitatory postsynaptic potentials (fEPSPs) in acutely prepared hippocampal slices from control mice and those expressing BlgC in the DG (Figure 2A). The input/output (I/O) curves and paired-pulse ratio (PPR) at MPP-DG synapses showed no significant difference between BlgC and control groups (Figures 2B,C), indicating that expression of BlgC does not affect basal synaptic properties. In addition, synaptic responses remained unchanged after 4 min of LED illumination (480 ± 15 nm, 1.5 mW) (Figure 2D), indicating that there is no immediate effect of cGMP on basal synaptic transmission.

### Postsynaptic cGMP enhances long-term potentiation

We next examined whether photoactivation of BlgC has any effect on LTP at MPP-DG synapses. To relieve the strong endogenous inhibition at dentate granule dendrites and maximize synaptic plasticity (Wigstrom and Gustafsson, 1983; Nguyen and Kandel, 1996), we applied strong tetanic stimulation (tetanus: 4 × 100 Hz) in the presence of 10 μM bicuculline to block GABA_A_ receptor-mediated inhibition (Saab et al., 2009). Immediately prior to induction, control or BlgC-expressing hippocampal slices received either 5 min of photostimulation (+Light) or no light (−Light) (Figures 2E,F). In the control mice, the light did not affect the extent of LTP (Figure 2E right). However, upon photostimulation of BlgC in combination with tetanic stimulation of the MPP, there was enhanced LTP that persisted for the additional 90-min recording period (Figure 2F). An analysis of the paired-pulse ratio (PPR), a measure of the probability of presynaptic transmitter release, was not significantly altered following LTP induction either in the absence or presence of BlgC and regardless of photostimulation (Figure 2G). The data show that tetanic stimulation in the presence of elevated postsynaptic cGMP significantly enhances LTP at MPP-DG synapses.

### Optogenetic cGMP generation in hippocampal DG neurons of freely behaving mice

**Figure 3:**
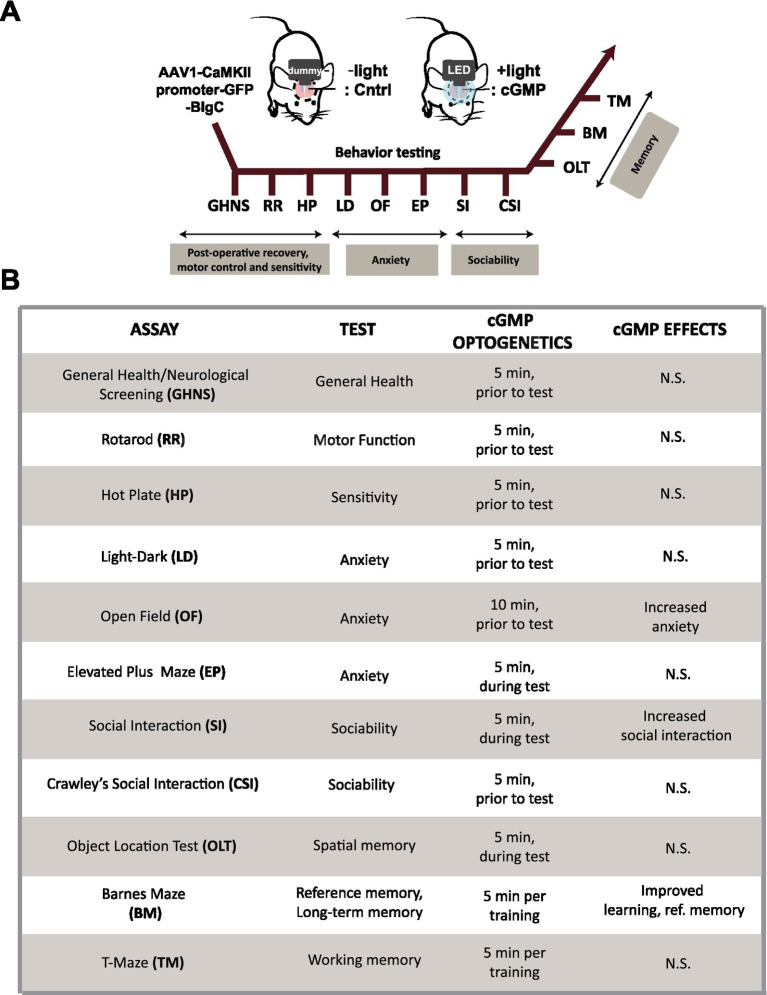
Schematic of mouse behavioral analysis. **(A)** Schematic and time course of the mouse behavioral tests performed using BlgC-expressing mice surgically equipped with LED and dummy implants for hippocampal photostimulation of DG BlgC. The mice were photoactivated for 5–10 min immediately prior to the behavioral tests (cGMP). **(B)** List of the mouse behavioral test battery. Summary of the mouse behavioral test battery showing the effect of photoactivated cGMP production on memory, anxiety and sociability.

To evaluate the effect of cGMP on a battery of mouse behavioral tests, we surgically implanted BlgC-expressing mice with either (i) a bilateral fiber optic-coupled and wireless LED system to deliver light to the DG that we refer to as the “cGMP” group or (ii) a control (Cntrl) group with “dummy” optic fiber which produces no light but exhibits the same physical properties (Figure 3A, upper). After a 2-week recovery following surgery, we carried out a battery of behavioral tests including general health and neurological screening, anxiety-like behavior, social interaction, and memory tests (Figure 3A, lower). We performed general health and neurological screening (GHNS), rotarod (RR), and hotplate (HP) test to determine the health condition and sensory/motor function of the animals (Supplementary Figure S2). The GHNS indicated no obvious abnormalities in general health, gross appearance, or nerve reflexes in the injected mice (Supplementary Figure S2A white bars). In the RR (Supplementary Figure S2B white bars) and HP tests (Supplementary Figure S2C white bars), the latency to fall and response to noxious temperature were indicative of good motor function and pain sensitivity in these mice. To examine the effect of cGMP on mouse condition and performance, we performed a 5-min BlgC photostimulation prior to the screen (Supplementary Figures S2A–C black bars, cGMP). The photostimulation of BlgC in hippocampal DG neurons did not show any effect on the basal performance in the mice, indicating that there is no significant effect of cGMP on the general health condition, motor function, and nociception of the mice.

### Effect of cGMP in hippocampal DG neurons on mouse behavior

**Figure 4:**
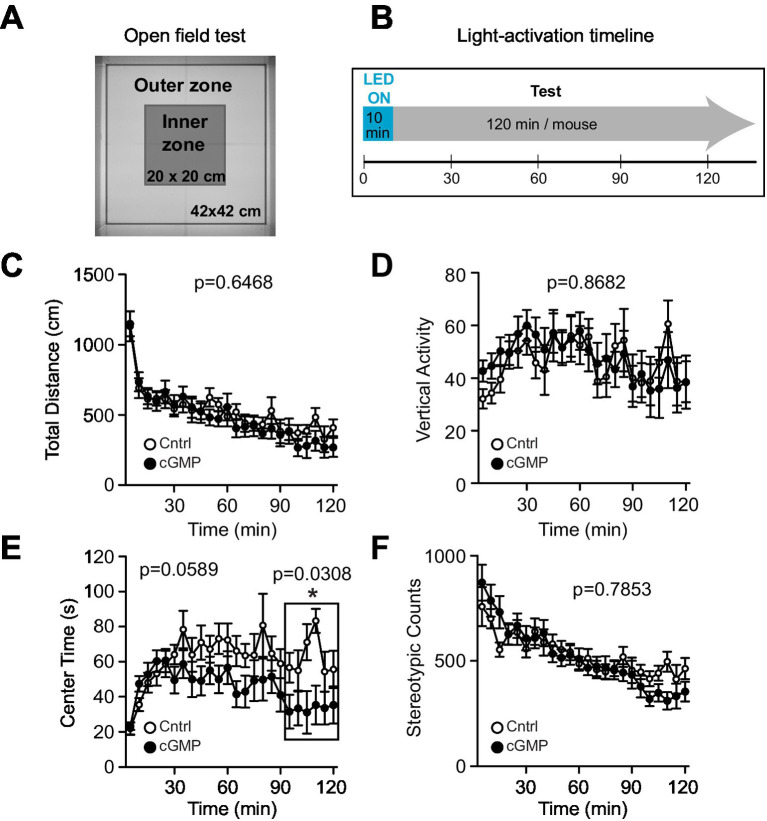
Open Field (OF) test. **(A)** Schematic of OF test. **(B)** Light (or mock) activation timeline during OF test. **(C-F)** Control (Cntrl) and photostimulated BlgC (cGMP) mice showed no significant differences in the total distance travelled **(C)**, Vertical activity **(D)**, Center time **(E)** and Stereotypic counts **(F)**. During the last 30 min of the OF test, cGMP mice spent significantly less time in the center of the arena compared with control (Cntrl) mice (E, in the frame). Cntrl *n* = 10, cGMP *n* = 13. Data are mean ± SEM. Statistical analysis was performed using a two-way repeated measures ANOVA.

**Figure 5:**
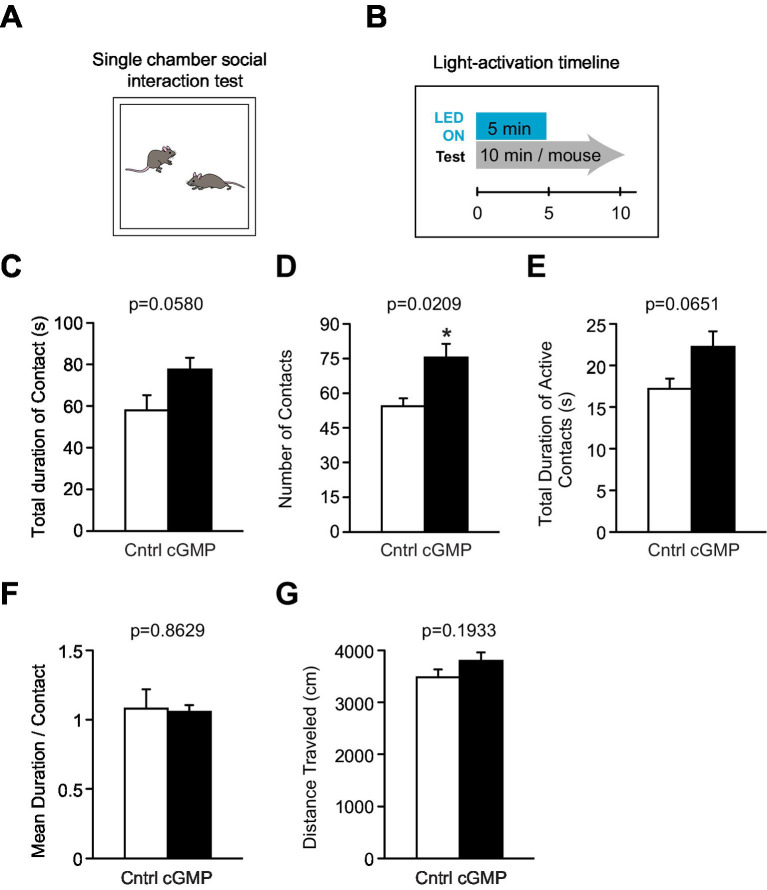
Single chamber Social Interaction (SI) test. **(A)** Schematic of SI test. **(B)** Light (or mock) activation timeline during SI test. (**C–G**) DG BlgC mice receiving photostimulation (cGMP) showed a significant increase in the total number of contacts **(D)** with a stranger mouse compared to controls. There were no significant differences between the two groups in the total duration of contact **(C)** and active contacts **(E)**, mean duration per contact **(F)**, and total distance traveled **(G)**. Cntrl *n* = 5 pairs, cGMP *n* = 7 pairs. Data are mean ± SEM. Statistical analysis was performed using an unpaired *t*-test.

**Figure 6:**
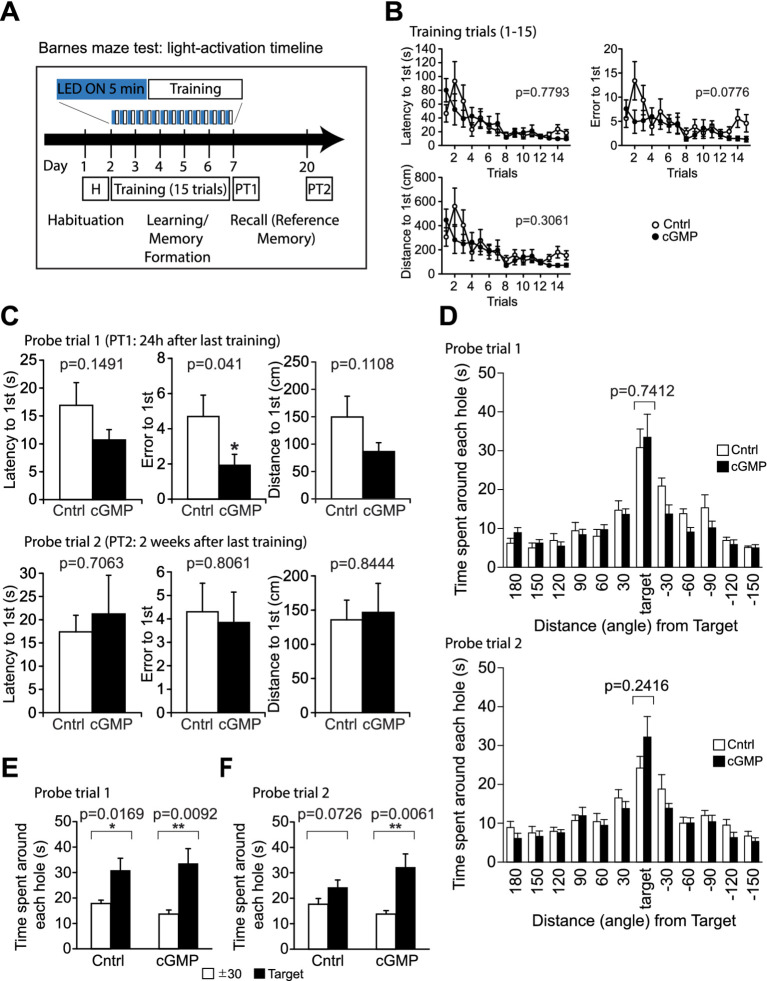
Barnes Maze (BM) test. **(A)** Schematic of light (or mock) activation timeline during BM test. During trials 1–15, mice are trained to learn the location of the escape box; learning is measured as latency(s), distance travelled (cm) and error to target hole. **(B)** DG cGMP mice did not show significant differences in the latency, error and distance to find the escape hole in the training session. **(C, D)** In probe test 1 (PT1), DG cGMP mice show a significantly lower error, but no significant differences in the time spent around the target escape hole. In probe test 2 (PT2), cGMP mice did not show any differences in the latency, error or distance to find the escape hole, and the time spent around the target escape hole. **(E-F)** 24 h **(E)** and 2 weeks **(F)** after the last training trial, mice were subjected to the probe tests (PT1 and PT2, respectively) and allowed to explore the arena for 3 min with the escape box removed. Total time spent around each hole was recorded. Cntrl *n* = 10, cGMP *n* = 13. Data are mean ± SEM. Statistical analysis was performed using a two-way repeated ANOVA (training session: B), and an unpaired *t*-test **(C, D)** and a paired *t*-test **(E, F)**.

We then examined the effect of optogenetic cGMP manipulation on anxiety-like behavior, social behavior, learning, and memory. The overall results of the study are shown in Figure 3B and the data for each significant test appear in Figures 4–6 and the others in Supplementary Figures S2–S7. Among the test battery performed in this study, the tests for anxiety-like behavior which last a total of 10 min, such as the light-dark (LD) (Supplementary Figure S3) test and the elevated plus maze (EP) test (Supplementary Figure S4), did not reveal any effect of BlgC photoactivation (cGMP generation in DG neurons). However, the open field (OF) test (Figure 4) showed that the cGMP group spent an increasingly reduced time in the center of the arena with stay time (Figure 4E), such that a statistically significant increase in anxiety-like behavior persisted at the end of the 120 min of observation. The single chamber social interaction (SI) test also showed an effect in the group receiving optogenetic production of cGMP in DG neurons (Figure 5), consisting of an increase in the number of contacts (Figure 5D) that signifies increased sociability. However, using Crawley’s social interaction (CSI) test, the DG cGMP group had no significant difference in the time spent interacting with a caged stranger over an empty cage (Supplementary Figure S5), suggesting social novelty was not affected, and therefore, cGMP may be specifically involved in sociability.

In the object location test (OLT) (Supplementary Figure S6) and T-maze (TM) test (Supplementary Figure S7), there were no significant differences in performance between controls (Cntrl) and photoactivated (cGMP) mice. In addition, the Barnes maze (BM) test (Figure 6) was used to examine training-dependent reference memory, where we photostimulated BlgC-expressing mice for 5 min in each daily training (Figure 6A). During the training trials, the photostimulated BlgC mice (cGMP) did not show significant differences in the latency, error, and distance to find the escape hole compared to control mice (Cntrl) (Figure 6B). In probe test 1 (PT1), which was conducted 24 h after the last training, there were fewer errors in the cGMP mice (Figure 6C), suggesting the improvement of spatial reference memory retrieval. However, there were no significant differences in the time spent around the target escape hole between groups (Figure 6D). Both of the groups maintained a strong preference to the target hole compared to the adjunct holes (Figure 6E) in PT1. In contrast, during probe test 2 (PT2) which was performed 2 weeks after the last training session (Figures 6C,D,F), photostimulated cGMP mice did not show any differences in the latency, error, or distance to find the escape hole compared to control mice (Figure 6C). In addition, there was no differences in the time spent around the target escape hole between groups (Figure 6D). While the control mice lost the preference to the target hole, cGMP mice maintained the preference after 2 weeks (Figure 6F), suggesting that there is an effect of cGMP on the enduring retention of reference memory. Altogether, cGMP signaling was associated with increased sociability, improved long-term reference memory, possibly at the expense of increased anxiety-like behavior over the longer term.

## Discussion

In this study, we demonstrated that elevation of cGMP specifically within DG granule neurons is capable of enhancing LTP at MPP-DG synapses and modifying mouse behaviors. The latter included a late-onset anxiety-related behavior, improvement of a long-term reference memory, accuracy-like behavior, and increased social behavior. As short-term presynaptic plasticity was unaffected, the modulation of cGMP levels was most probably specific to postsynaptic neurons. These results further suggest that the postsynaptic cGMP actions on MPP-DG synaptic plasticity may contribute to shorter-term cognitive and sociability effects.

### Optogenetic manipulation of cGMP

To manipulate cGMP in neurons, we virally expressed photoactivatable guanylyl cyclase (BlgC) in the granule neurons of the hippocampal DG region. We prepared the brain slices and imaged the CaMKIIα-driven expression of GFP-tagged BlgC. Confocal imaging revealed that the mice expressed abundant levels of GFP within the dentate granule neurons including the mossy fiber axons, indicating the localized expression of BlgC within the DG granule neurons, but not the presynaptic perforant path fibers. We next validated photoactivation of the expressed BlgC. The lysates from the acute slices showed cGMP production upon delivery of blue light. To directly observe the synthesized cGMP by photoactivation of BlgC in living neurons, we coexpressed a cGMP FLIM probe (cGiYR) with the BlgC in living hippocampal organotypic cultured neurons and detected the FLIM change immediately after blue light stimulation. Expression of cAMP FLIM probe (REY) with BlgC did not show the FLIM change upon the light stimulation, indicating the increase of cGMP, but no detectable change of cAMP levels in the neurons.

### cGMP elevation enhances LTP in granule cells

The expression of BlgC in the granule neurons did not affect the baseline fEPSPs at MPP-DG synapses in the hippocampal slices, nor the paired-pulse ratio suggesting no changes in the probability of release. Photostimulation of BlgC also did not change the fEPSPs during recording of the basal synaptic responses. We next evaluated DG LTP induced by tetanic activation of MPP fibers in the presence of the GABA_A_-receptor blocker (10 μM bicuculline). Using this system, we demonstrated that the short blue light stimulation of photoactivatable BlgC coincident with the tetanic stimulation enhanced the level of evoked LTP. However, the presynaptic function was unaffected, indicating that when postsynaptic cGMP is elevated during high-frequency synaptic activity (tetanic stimulation), there is an enhanced postsynaptic potentiation of synaptic strength that persists for at least 90 min. This interesting feature of cGMP-dependent enhancement of LTP at MPP-DG indicates that cGMP has a selective impact during conditions that enable synaptic plasticity. Indeed, it has been previously shown that cGMP signaling pathways are involved in LTP in hippocampus CA1 neurons, where NO-GC-PKG signaling has been reported as a retrograde messenger system from post- to presynaptic terminal for the induction of LTP, suggesting a presynaptic cGMP function (O’Dell et al., 1991; Zhuo et al., 1994; Arancio et al., 1996). NO has been shown to be involved in LTP induction at the MPP-DG synapse but the signaling mechanism may not involve PKG, as determined by using pharmacological inhibition assays (Wu et al., 1997, 1998). Therefore, the LTP-enhancing pathways downstream of cGMP remain to be determined.

### cGMP elevation enhances mouse behaviors

We employed a wireless LED with fiber optics implant system for photoactivation of BlgC in the DG neurons of freely behaving mice and simultaneously recorded the behavioral changes. BlgC photostimulation did not affect the general health condition or motor function of the animals.

In the behavioral test battery, the shorter behavioral tests of anxiety (10 min tests) such as LD and EP did not reveal any significant changes following photostimulation of BlgC. In the OF test, we photostimulated BlgC two times longer (10 min) than in other behavioral tests (LD and EP), but the mice did not show any increase in anxiety-like behavior during the initial 90 min of testing.

Upon exposure to the open field, compared to the BlgC group, the control group began to explore the center of the arena with time. The simplest interpretation is that control mice gradually overcome their fear or anxiety and begin to explore the most exposed part of the arena.

Interestingly, in the latter portion of the OF testing (90–120 min), BlgC photostimulated mice continued to avoid the center of the arena, which may reflect increased anxiety-like behavior. Both the NO/cGMP pathway and the hippocampus are implicated in stress responses and anxiety (Spolidorio et al., 2007; Calixto et al., 2010; Ghasemi et al., 2022; Shi et al., 2023). However, it must be noted that in contrast to the OF test, there were no differences in the time spent in the bright section of the light-dark arena, possibly because this latter test is much shorter in duration (120 vs. 15 min, respectively). Similarly, the 10-min EP test did not reveal any differences in time spent in the open arms. Additional longer testing may be able to clarify whether stress, anxiety, or possibly improved spatial learning, which is the reason underlying the open field results.

Notably, cGMP is known to decrease with aging (Domek-Lopacinska and Strosznajder, 2010), and aging impairs cognitive function as measured in the Barnes maze for mice (Shoji et al., 2016; Shoji and Miyakawa, 2019). At the same time, aging increases center time stay in the OF test, especially in the latter half of the period (Shoji et al., 2016; Shoji and Miyakawa, 2019). In the present study, cGMP was increased locally in the dentate gyrus, and the phenomena observed in both the BM and OF test were opposite to the phenotype of the aged mice, in which cGMP is expected to be decreased. Whether the anxiety-like behavior observed in OF is associated with spatial memory is not clear; however, our results may suggest that the dentate gyrus and cGMP are involved in both anxiety-like behavior and spatial memory.

The excitatory entorhinal cortical perforant projections to the dentate gyrus (EC-DG) neurons are tightly involved in social memory (Leung et al., 2018). In the SI test (10-min duration) following photostimulation of BlgC in DG neurons, there was a rapid effect for cGMP in enhancement of social behavior. It is not clear why cGMP and control groups performed similarly in the CSI test that compares interactions with either a caged stranger mouse or familiar mouse, or empty cage. The role of cGMP could be specific to direct social interaction between mice.

As postsynaptic cGMP elevation enhanced LTP at the MPP-DG synapses, we examined performance in several learning and memory tasks during photoactivation of BlgC in DG neurons. In the memory test battery, the BlgC group receiving photostimulation did not show any significant differences on tasks including the OLT, training period of the BM, and the TM test, suggesting no immediate effect of cGMP in the learning and memory. However, in the OLT, even the control mice showed a strong memory of the object novel location, which could have made it difficult to detect any further improvements in the cGMP group. Using an OLT training protocol that generates weak memory in control mice may help to further evaluate the more immediate function of cGMP.

Interestingly, 24 h after the training in the BM test, the cGMP mice showed significantly improved performance result compared to controls. This learning and memory improvement after training with photostimulation of BlgC could be due to cumulative cGMP-dependent enhancement of synaptic potentiation during the trials. When we assessed mice 2 weeks after the training trial, the controls already lost the reference memory, but the cGMP group showed a maintenance of the preference memory. Thus, cGMP in the DG neurons could serve to enhance the retrieval and long-term retention or reference memory.

The observation that cGMP elevation only affected a few of the behavioral tasks may relate to the relative cognitive role of the dentate in each case. Some specific roles of the dentate include pattern separation (novelty detection) and episodic memory encoding, though there is controversy on its role in memory retrieval or recall (Hainmueller and Bartos, 2020; Borzello et al., 2023). In terms of elevated cGMP, a PDE5 inhibitor has been shown to facilitate long-term retention of inhibitory avoidance memory (Baratti and Boccia, 1999). In addition, PDE2 inhibition has also been shown to improve memory in a cGMP-dependent manner (Lueptow et al., 2016). Interestingly, cGMP/PKG was found to mediate early memory consolidation and early-phase LTP, whereas cAMP/PKA mediated the late consolidation of LTP (Bollen et al., 2014); there is also evidence suggesting distinct changes involving increases in surface GluA1- and/or GluA2-containing AMPARs in the two signaling pathways with respect to acquisition versus consolidation phases of memory (Argyrousi et al., 2020). Any specific cGMP-mediated increase in GluA1 could potentially relate to the incorporation of calcium-permeable AMPARs that have been shown to be essential in cAMP/PKA-dependent forms of long-lasting LTP (Park et al., 2018; Park et al., 2021).

Presynaptic effects of elevating cGMP have also been described (Boulton et al., 1994; Fieblinger et al., 2022). We did not find any evidence for presynaptic actions, which may be because cGMP was not elevated in the perforant path inputs. In which case, additional roles of presynaptic alterations in cGMP signaling on dentate-dependent behaviors cannot be excluded.

In summary, our optogenetic approach using a light-sensitive BlgC actuator allowed us to directly assess the role of DG cGMP elevations on synaptic function by electrophysiology and behavioral functions that included cognition, sociability, and anxiety. The results demonstrate that postsynaptic cGMP elevation can facilitate LTP at MPP synapses onto DG granule cells. In behaving mice, DG cGMP increased sociability, had a long-term anxiogenic action, and improved reference memory. In future work, the optogenetic BlgC actuator could, in principle, be used to study spatiotemporal aspects of cGMP functions in synaptic plasticity and learning and memory. By extension, the results suggest that methods to elevate cGMP specifically within dentate granule neurons may provide a means to modify brain function in various brain disorders.

## Data Availability

The raw data supporting the conclusions of this article will be made available by the authors, without undue reservation.

## Supplementary material

SUPPLEMENTARY FIGURE S1 Detection of BlgC photoactivation. **(A)** A FRET/FLIM probe (cGiYR) based on cGMP binding domains in PKG for cGMP detection. **(B)** Dose-dependency of cGiYR FLIM signal for 8-Br-cGMP with an EC_50_ = 0.48 μM (*n* = 3; Hill fit). **(C)** Quantification of the fluorescent lifetime: baseline, +100 μM 8-Br-cGMP, and YFP alone. cGiYR (*n* = 3), cGiYR + cGMP (*n* = 3), and YFP (*n* = 10). Tukey’s test. **(D)** Schematic showing blue light activation of BLUF (blue-light sensing using flavins) coupled to GC (BlgC) and detection of the produced cGMP by cGiYR. **(E)** Detection of cGMP increase in a primary dendritic region including spine, evoked by BlgC photoactivation in living neurons within a cultured organotypic hippocampus slice. Left: The cGMP pseudo-color cGiYR FLIM images before and after photostimulation (414 ± 21 nm mercury arc lamp, 11 mW, 60 s). Right: Time-course of the fluorescence lifetime change in neurons expressing either cGiYR with or without BlgC (+/−BlgC). A cAMP probe (REY) is also expressed with BlgC (REY + BlgC) as a control. +BlgC (*n* = 16), −BlgC (*n* = 11), and REY + BlgC (*n* = 11). All data are mean ± SEM.

SUPPLEMENTARY FIGURE S2 **(A)** General health and neurological screening (GHNS) test. Measurements of body weight, body temperature and strength using grip and wire hang methods showed no significant abnormalities in mice 2 weeks after surgery. **(B,C)** RotaRod (RR) test **(B)** and hot plate (HP) test **(C)** showed no abnormalities in motor function and sensitivity of mice. Cntrl *n* = 10, cGMP *n* = 13. Data are mean ± SEM. Statistical analysis was performed using an unpaired *t*-test **(A,C)** and a two-way repeated measures ANOVA **(B)**.

SUPPLEMENTARY FIGURE S3 Light-dark (LD) test. **(A)** Schematic of LD test. **(B)** Light (or mock) activation timeline during LD test. **(C–F)** Control (Cntrl) and photostimulated BlgC (cGMP) mice both showed a preference for the dark compartment (**C**, light and dark), but no significant group differences in distance travelled **(C)**, stay time in light **(D)**, number of transitions between compartments **(E)** or latency to light **(F)**. Cntrl *n* = 10, cGMP *n* = 14. Data are mean ± SEM. Statistical analysis was performed using an unpaired *t*-test.

SUPPLEMENTARY FIGURE S4 Elevated plus maze (EP) test. **(A)** Schematic of EP test. **(B)** Light (or mock) activation timeline during EP test. **(C–F)** Control (Cntrl) and photostimulated BlgC (cGMP) mice showed no significant differences in the number of entries into open arms **(C)**, percent of entries into open arms **(D)**, total distance travelled **(E)** or the percent of time spent in open arms **(F)** of the elevated maze. Cntrl *n* = 10, cGMP *n* = 12. Data are mean ± SEM. Statistical analysis was performed using an unpaired *t*-test.

SUPPLEMENTARY FIGURE S5 Crawley’s social interaction (CSI) test. **(A)** Schematic of CSI test. **(B)** Light (or mock) activation timeline during CSI test. **(C–F)** Control and photostimulated BlgC (cGMP) mice show a significant preference for the cage with a stranger mouse compared to empty cage **(C)**. The social preference index was calculated for each group [time exploring stranger cage (time exploring empty cage + time exploring stranger cage)] and showed no significant difference between cGMP and control mice **(D)**. Similarly, both groups showed preference for the novel stranger mouse **(E)**, but no significant difference in social novelty preference index between the two groups **(F)**. Cntrl *n* = 10, cGMP *n* = 12. Data are mean ± SEM. Statistical analysis was performed using a paired *t*-test **(C,E)** and an unpaired *t*-test **(D,F)**.

SUPPLEMENTARY FIGURE S6 Object location test (OLT). **(A)** Schematic of OLT. **(B)** Light (or mock) activation timeline during OLT. During familiarization phase, mice are introduced to the original configuration of two identical objects, and cGMP signaling is achieved by light activation of BlgC in the DG. Data shown in the following bar plots is from the first 5-min averages of test phase, where one object has been displaced (object in novel location, black bars) and mice are placed back into the arena 5 min later. **(C–G)** Both control and photostimulated BlgC (cGMP) mice show a preference for novelty based on time spent around displaced object **(C,D)** and number of entries to the novel object region **(E,F)**. The discrimination index: time around novel object/(time around novel object + time around familiar object) shows no significant difference between control and cGMP groups **(G)**. Cntrl *n* = 10, cGMP *n* = 12. Data are mean ± SEM. Statistical analysis was performed using a paired *t*-test **(C–F)** and an unpaired *t*-test for discrimination index **(G)**.

SUPPLEMENTARY FIGURE S7 Forced alternation T-maze test (TMFA). **(A)** Schematic of TMFA test. **(B)** Light (or mock) activation timeline during TMFA test. After a 1–2-weeks food restriction phase, mice reach ~80–85% of their original body weight and are considered motivated enough for the food reward (sucrose pellets). Pellet is delivered to alternating arms while the correct responses, distance and latency of each mouse is recorded. During delay trials, a 3, 10, 30, or 60 s delay is inserted between each arm alteration task. **(C)** There was no significant difference in the correct responses (%), latency (s) or distance travelled between mice stimulated to produce cGMP (cGMP, black bars) and control mice (Cntrl, white bars). **(D)** When a delay was introduced between each arm alternation (3–60 s), there was no significant difference in the percentage of correct responses between control and cGMP mice. Cntrl *n* = 10, cGMP *n* = 9. Data are mean ± SEM. Statistical analysis was performed using a two-way repeated measures ANOVA **(C)** and an unpaired *t*-test **(D)**.
