# Supplementary figures and images for: Optogenetic elevation of postsynaptic cGMP in the hippocampal dentate gyrus enhances LTP and modifies mouse behaviors
Source: Front Mol Neurosci. 2024 Nov 26;17:1479360. doi: 10.3389/fnmol.2024.1479360 (PMC11629205; doi:10.3389/fnmol.2024.1479360)

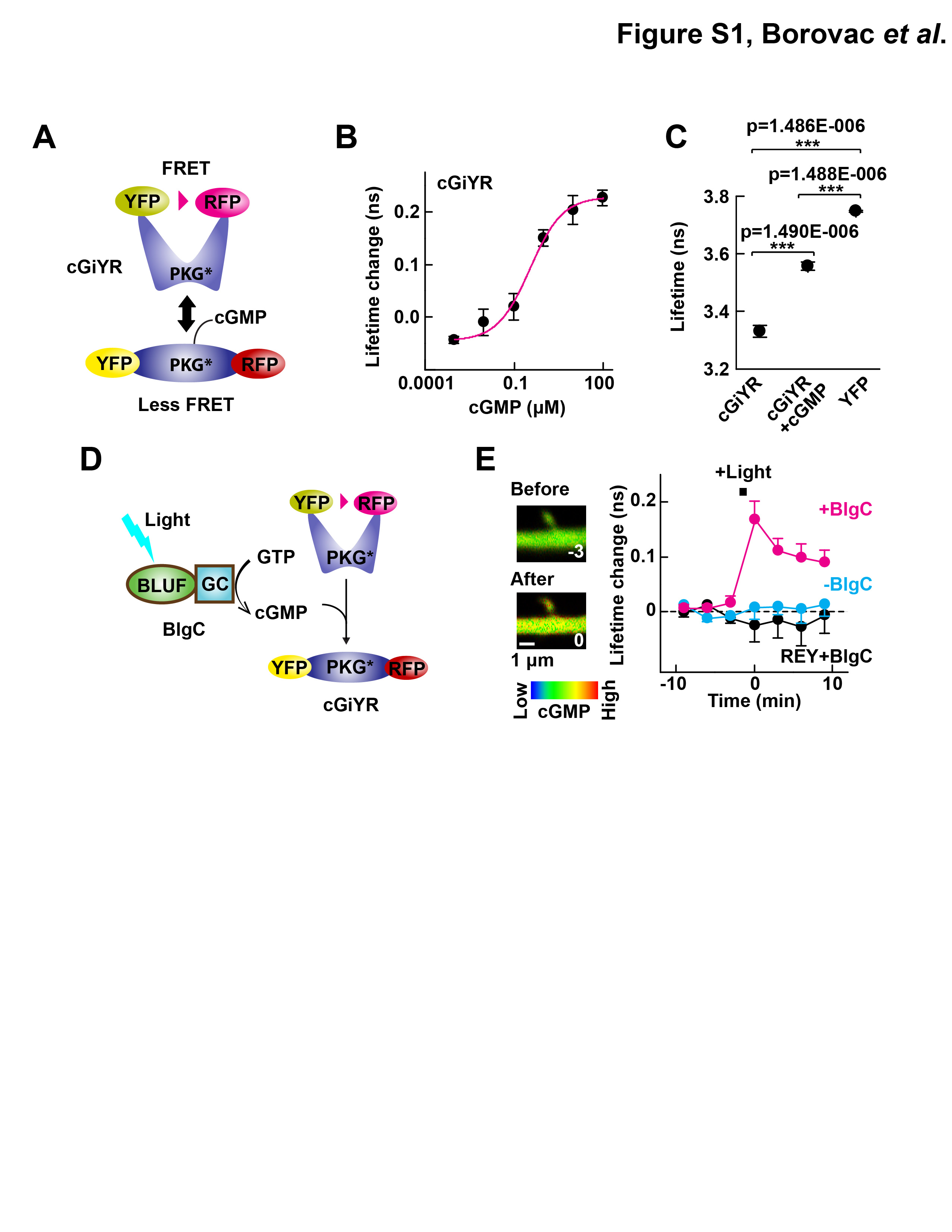

Supplement: SUPPLEMENTARY FIGURE S1 — Detection of BlgC photoactivation. (A) A FRET/FLIM probe (cGiYR) based on cGMP binding domains in PKG for cGMP detection. (B) Dose-dependency of cGiYR FLIM signal for 8-Br-cGMP with an EC50 = 0.48 μM (n = 3; Hill fit). (C) Quantification of the fluorescent lifetime: baseline, +100 μM 8-Br-cGMP, and YFP alone. cGiYR (n = 3), cGiYR + cGMP (n = 3), and YFP (n = 10). Tukey’s test. (D) Schematic showing blue light activation of BLUF (blue-light sensing using flavins) coupled to GC (BlgC) and detection of the produced cGMP by cGiYR. (E) Detection of cGMP increase in a primary dendritic region including spine, evoked by BlgC photoactivation in living neurons within a cultured organotypic hippocampus slice. Left: The cGMP pseudo-color cGiYR FLIM images before and after photostimulation (414 ± 21 nm mercury arc lamp, 11 mW, 60 s). Right: Time-course of the fluorescence lifetime change in neurons expressing either cGiYR with or without BlgC (+/−BlgC). A cAMP probe (REY) is also expressed with BlgC (REY + BlgC) as a control. +BlgC (n = 16), −BlgC (n = 11), and REY + BlgC (n = 11). All data are mean ± SEM. [file Image_1.jpg]

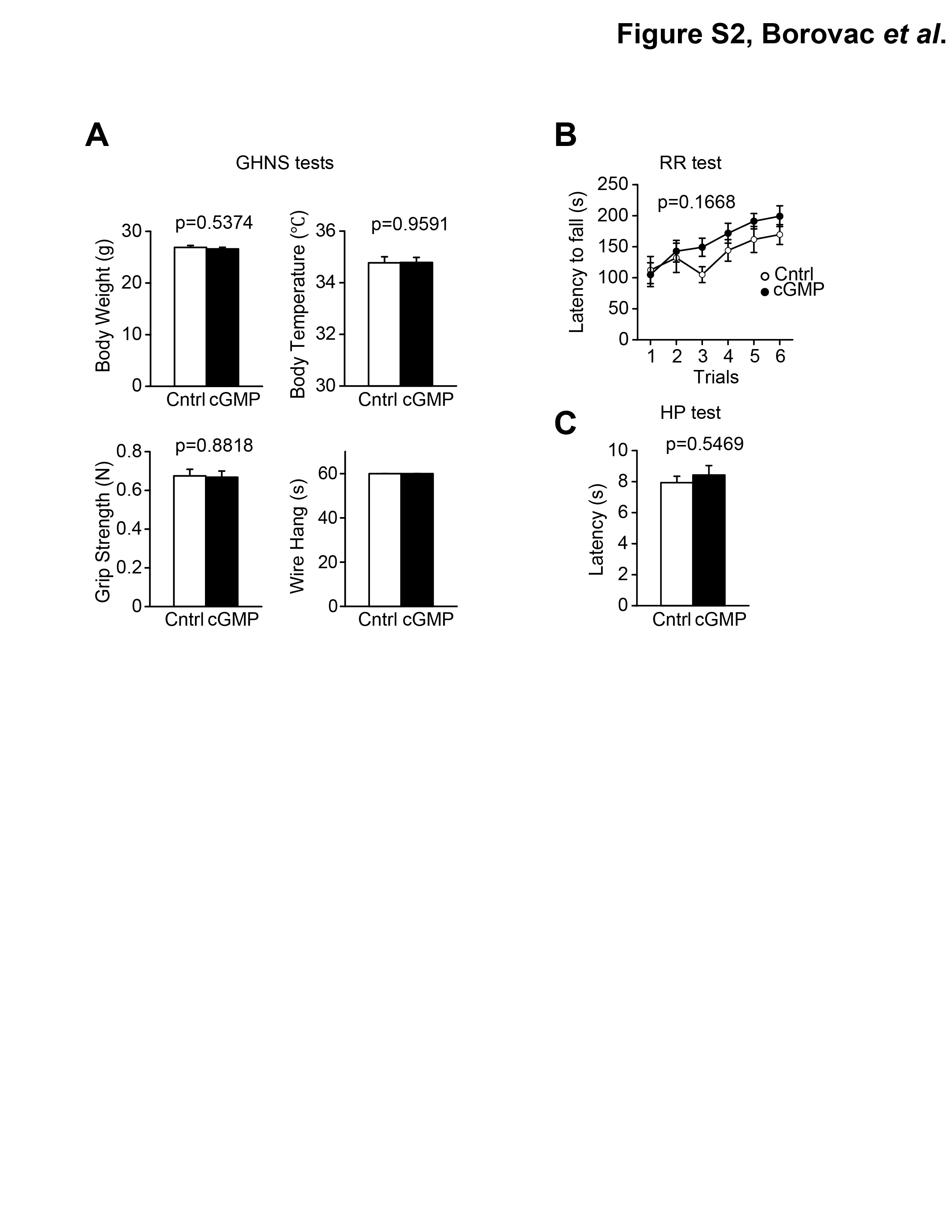

Supplement: SUPPLEMENTARY FIGURE S2 — (A) General health and neurological screening (GHNS) test. Measurements of body weight, body temperature and strength using grip and wire hang methods showed no significant abnormalities in mice 2 weeks after surgery. (B,C) RotaRod (RR) test (B) and hot plate (HP) test (C) showed no abnormalities in motor function and sensitivity of mice. Cntrl n = 10, cGMP n = 13. Data are mean ± SEM. Statistical analysis was performed using an unpaired t-test (A,C) and a two-way repeated measures ANOVA (B). [file Image_2.jpg]

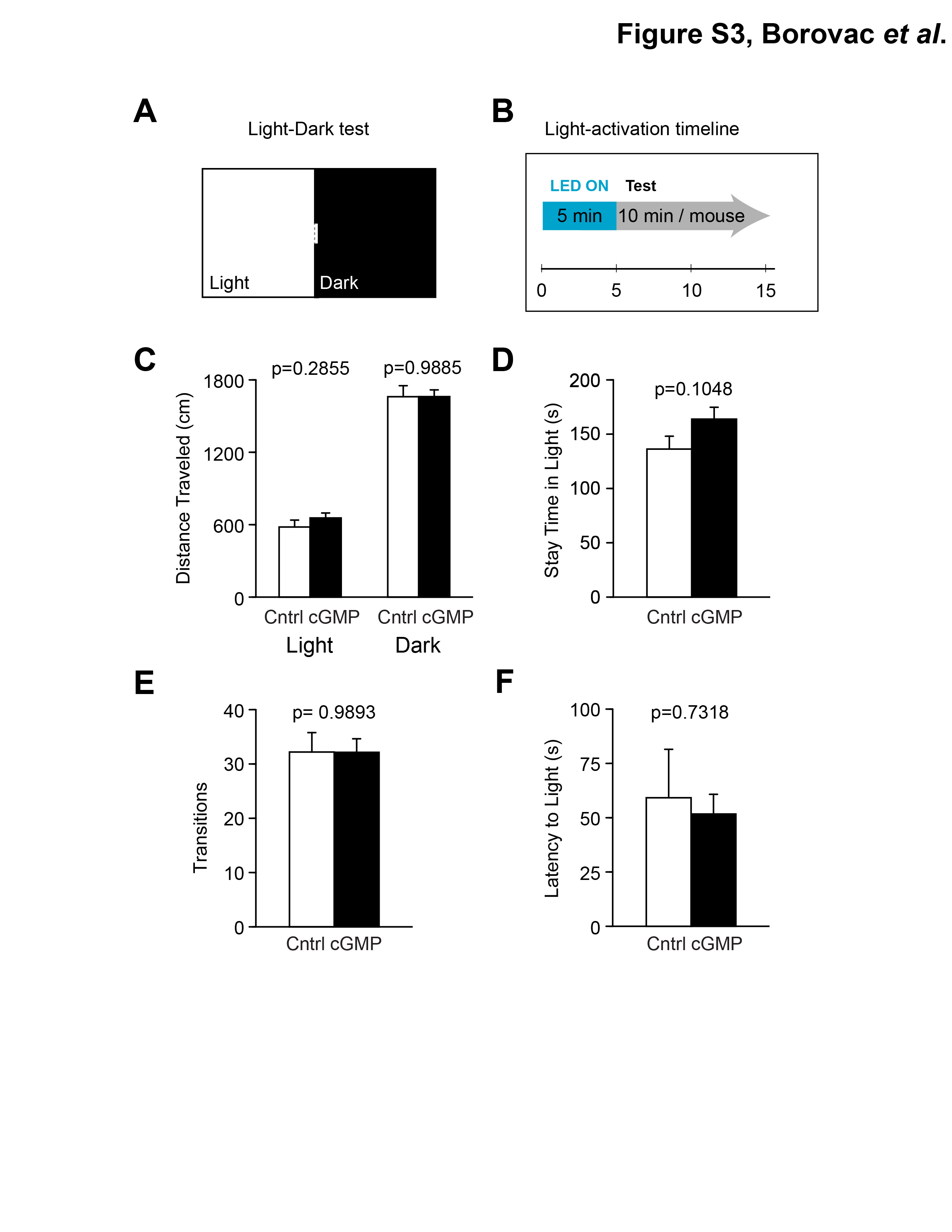

Supplement: SUPPLEMENTARY FIGURE S3 — Light-dark (LD) test. (A) Schematic of LD test. (B) Light (or mock) activation timeline during LD test. (C–F) Control (Cntrl) and photostimulated BlgC (cGMP) mice both showed a preference for the dark compartment (C, light and dark), but no significant group differences in distance travelled (C), stay time in light (D), number of transitions between compartments (E) or latency to light (F). Cntrl n = 10, cGMP n = 14. Data are mean ± SEM. Statistical analysis was performed using an unpaired t-test. [file Image_3.jpg]

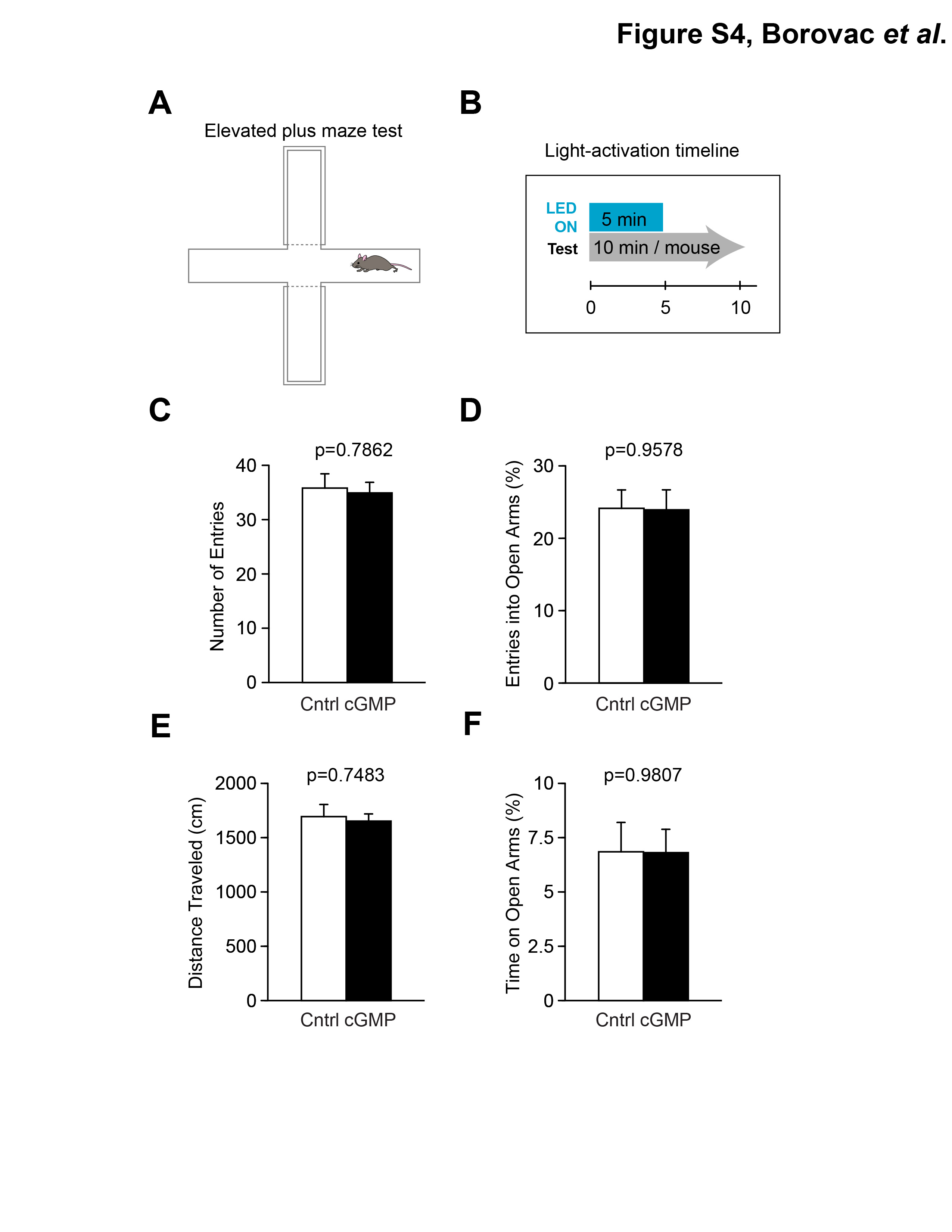

Supplement: SUPPLEMENTARY FIGURE S4 — Elevated plus maze (EP) test. (A) Schematic of EP test. (B) Light (or mock) activation timeline during EP test. (C–F) Control (Cntrl) and photostimulated BlgC (cGMP) mice showed no significant differences in the number of entries into open arms (C), percent of entries into open arms (D), total distance travelled (E) or the percent of time spent in open arms (F) of the elevated maze. Cntrl n = 10, cGMP n = 12. Data are mean ± SEM. Statistical analysis was performed using an unpaired t-test. [file Image_4.jpg]

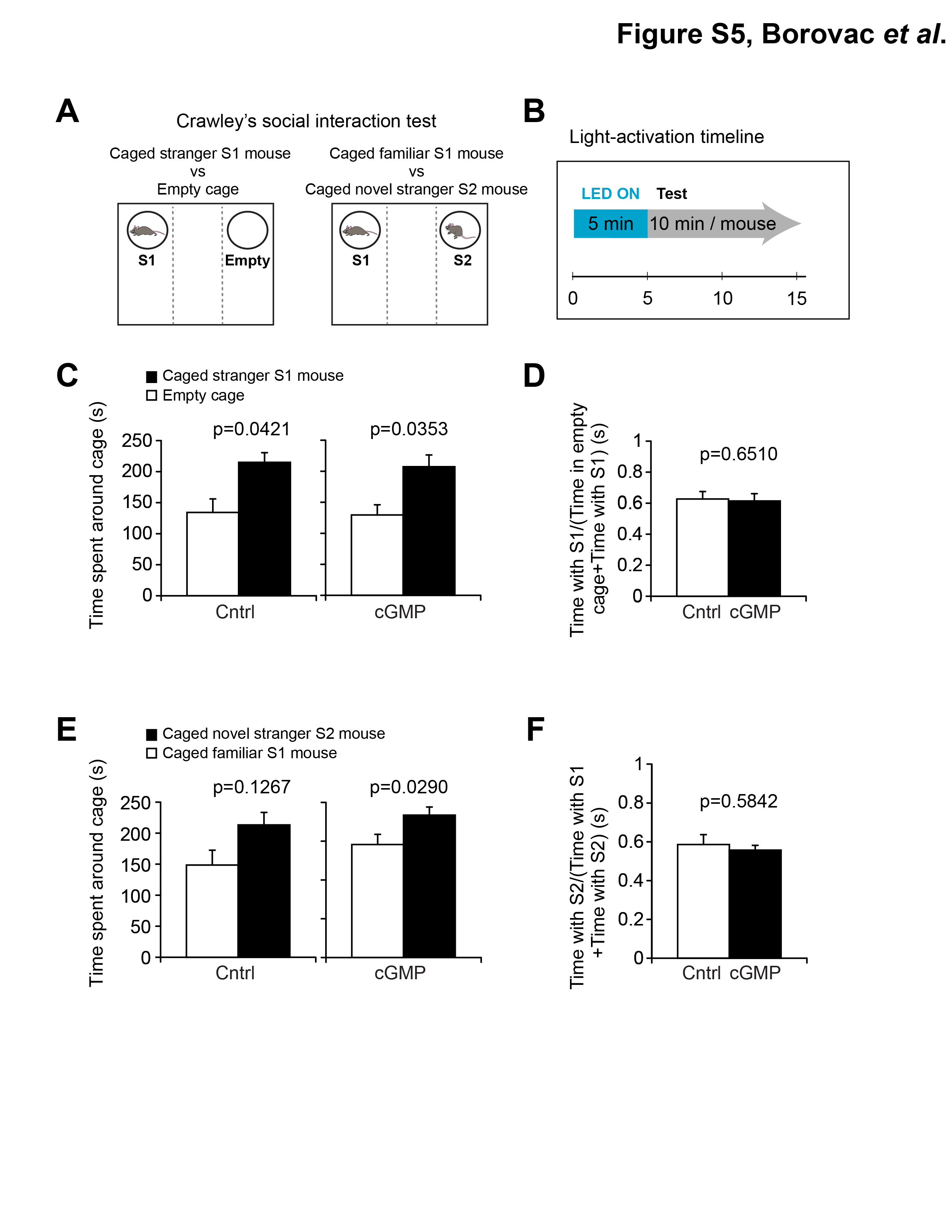

Supplement: SUPPLEMENTARY FIGURE S5 — Crawley’s social interaction (CSI) test. (A) Schematic of CSI test. (B) Light (or mock) activation timeline during CSI test. (C–F) Control and photostimulated BlgC (cGMP) mice show a significant preference for the cage with a stranger mouse compared to empty cage (C). The social preference index was calculated for each group [time exploring stranger cage (time exploring empty cage + time exploring stranger cage)] and showed no significant difference between cGMP and control mice (D). Similarly, both groups showed preference for the novel stranger mouse (E), but no significant difference in social novelty preference index between the two groups (F). Cntrl n = 10, cGMP n = 12. Data are mean ± SEM. Statistical analysis was performed using a paired t-test (C,E) and an unpaired t-test (D,F). [file Image_5.jpg]

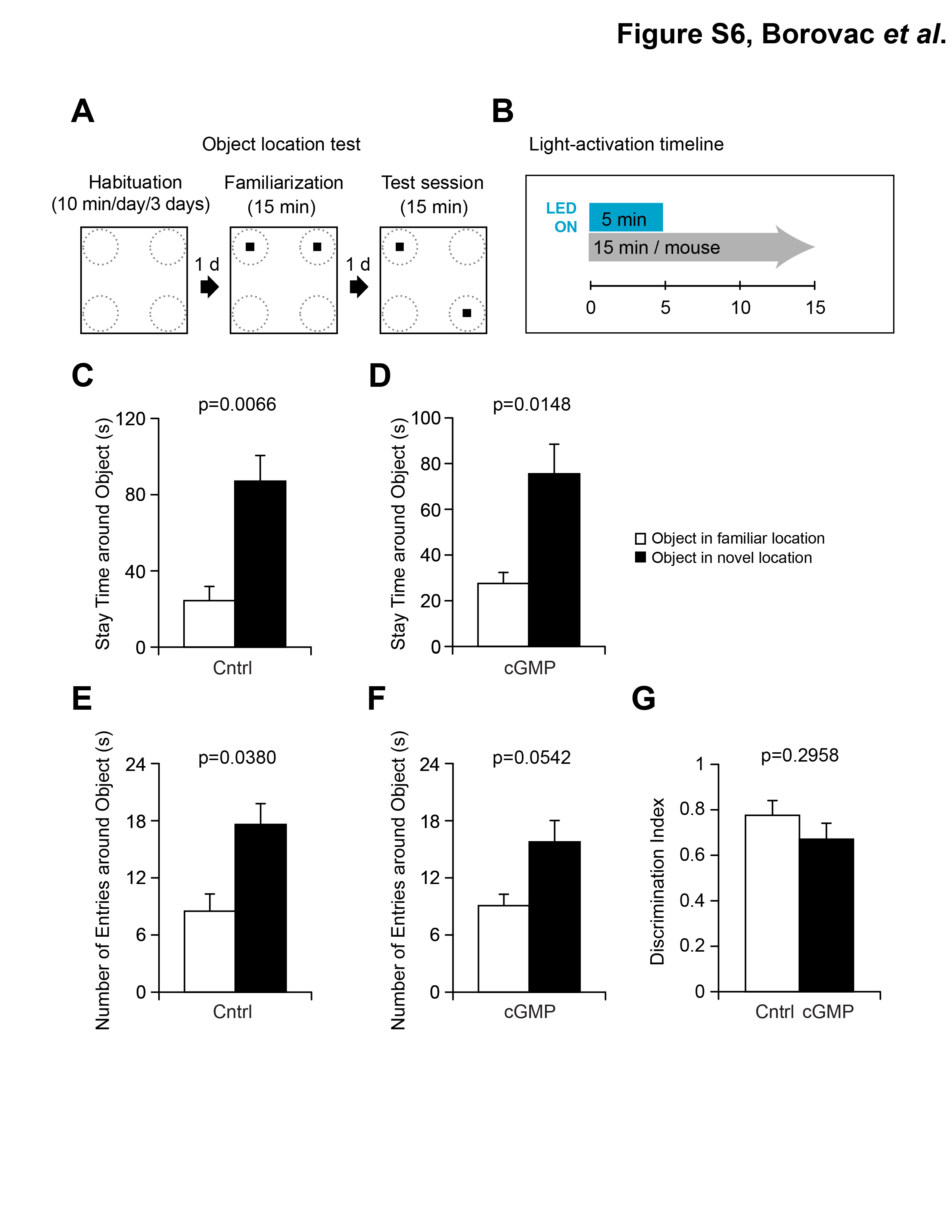

Supplement: SUPPLEMENTARY FIGURE S6 — Object location test (OLT). (A) Schematic of OLT. (B) Light (or mock) activation timeline during OLT. During familiarization phase, mice are introduced to the original configuration of two identical objects, and cGMP signaling is achieved by light activation of BlgC in the DG. Data shown in the following bar plots is from the first 5-min averages of test phase, where one object has been displaced (object in novel location, black bars) and mice are placed back into the arena 5 min later. (C–G) Both control and photostimulated BlgC (cGMP) mice show a preference for novelty based on time spent around displaced object (C,D) and number of entries to the novel object region (E,F). The discrimination index: time around novel object/(time around novel object + time around familiar object) shows no significant difference between control and cGMP groups (G). Cntrl n = 10, cGMP n = 12. Data are mean ± SEM. Statistical analysis was performed using a paired t-test (C–F) and an unpaired t-test for discrimination index (G). [file Image_6.jpg]

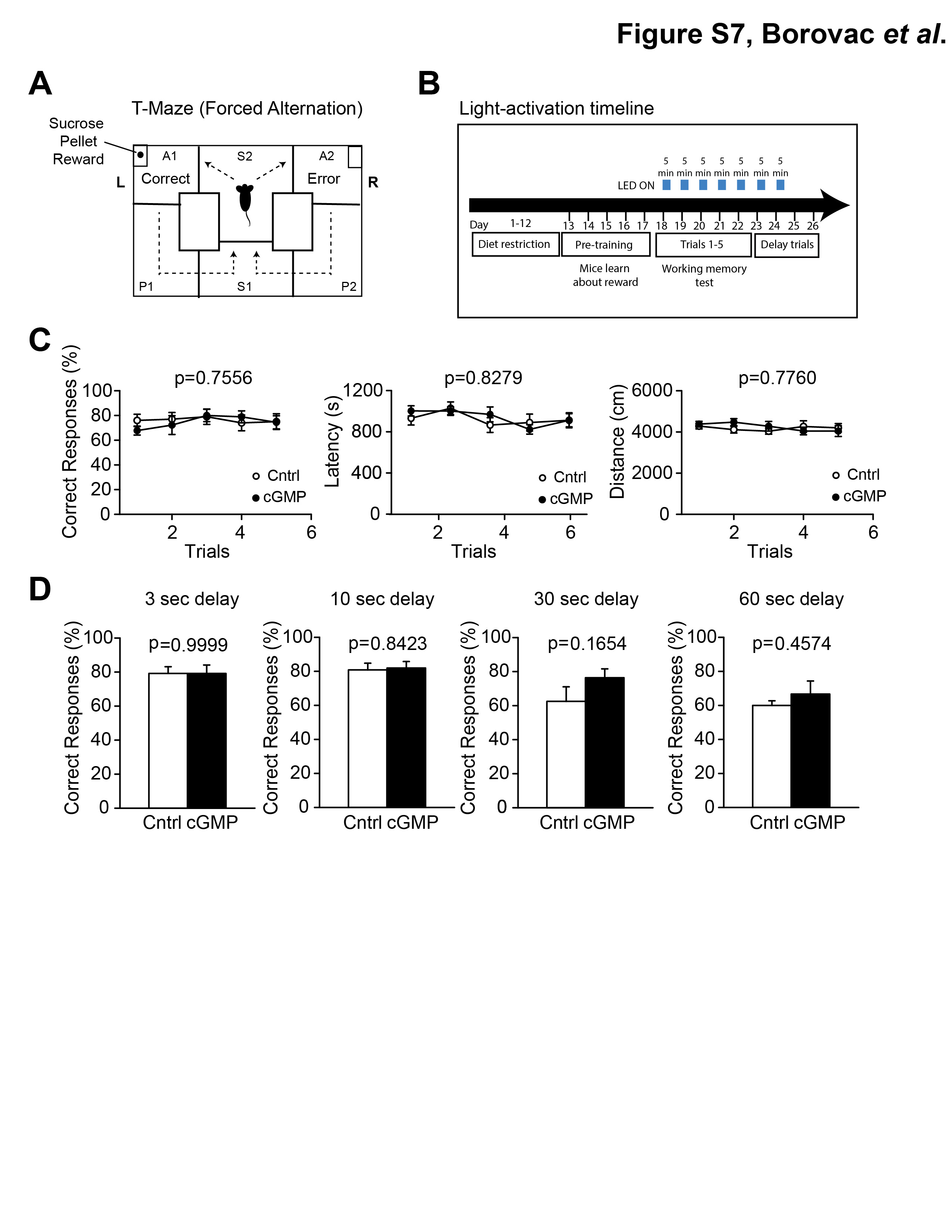

Supplement: SUPPLEMENTARY FIGURE S7 — Forced alternation T-maze test (TMFA). (A) Schematic of TMFA test. (B) Light (or mock) activation timeline during TMFA test. After a 1–2-weeks food restriction phase, mice reach ~80–85% of their original body weight and are considered motivated enough for the food reward (sucrose pellets). Pellet is delivered to alternating arms while the correct responses, distance and latency of each mouse is recorded. During delay trials, a 3, 10, 30, or 60 s delay is inserted between each arm alteration task. (C) There was no significant difference in the correct responses (%), latency (s) or distance travelled between mice stimulated to produce cGMP (cGMP, black bars) and control mice (Cntrl, white bars). (D) When a delay was introduced between each arm alternation (3–60 s), there was no significant difference in the percentage of correct responses between control and cGMP mice. Cntrl n = 10, cGMP n = 9. Data are mean ± SEM. Statistical analysis was performed using a two-way repeated measures ANOVA (C) and an unpaired t-test (D). [file Image_7.jpg]
